# 1611. Real-World Effectiveness and Tolerability of Bictegravir/Emtricitabine/Tenofovir Alafenamide (B/F/TAF) in Treatment-Experienced People With HIV and a History of Antiretroviral Drug Resistance Mutations

**DOI:** 10.1093/ofid/ofad500.1446

**Published:** 2023-11-27

**Authors:** Benoit Trottier, Fabrice Bonnet, Miguel Garcia-Deltoro, Massimo Andreoni, Marta Boffito, Berend J van Welzen, Dan Turner, Sam McConkey, Dai Watanabe, Po-Liang Lu, Alper Gündüz, David Thorpe, Michelle L D’Antoni, Tali Cassidy, Andrea Marongiu, Amy R Weinberg, Richard Haubrich, Stefan Scholten

**Affiliations:** Clinique de medecine urbaine du Quartier Latin, Montreal, Quebec, Canada; CHU de Bordeaux, Hôpital Saint André, Bordeaux, Aquitaine, France; Concorcio Hospital General Universitario de Valencia, Valencia, Comunidad Valenciana, Spain; University Tor Vergata, Rome, Lazio, Italy; Chelsea and Westminster Hospital NHS Foundation Trust and Imperial College London, London, England, United Kingdom; University Medical Center Utrecht, Utrecht, Utrecht, Netherlands; Crusaid Kobler AIDS Center, Sackler Faculty of Medicine, Tel Aviv University, Tel Aviv, Tel Aviv, Israel; RCSI University of Medicine and Health Sciences, Dublin, Dublin, Ireland; National Hospital Organization Osaka National Hospital, Osaka, Osaka, Japan; Kaohsiung Medical University, Kaohsiung, Kaohsiung, Taiwan; Basaksehir Cam and Sakura City Hospital, Istanbul, Istanbul, Turkey; Gilead Sciences Europe Ltd, Stockley Park, England, United Kingdom; Gilead Sciences, Inc., Foster City, California; Gilead Sciences Europe Ltd, Stockley Park, England, United Kingdom; Gilead Sciences Europe Ltd, Stockley Park, England, United Kingdom; Gilead Sciences, Inc., Foster City, California; Gilead Sciences, Foster City, California; Medical practice Hohenstaufenring, Cologne, Germany, Köln, Nordrhein-Westfalen, Germany

## Abstract

**Background:**

BICSTaR is an ongoing, multinational, observational cohort study evaluating the real-world effectiveness and safety of B/F/TAF in antiretroviral therapy (ART) treatment-naïve and treatment-experienced (TE) people with HIV.

**Methods:**

This analysis of BICSTaR included TE virologically suppressed people with HIV who had started B/F/TAF in clinical practice with/without present/past evidence of HIV drug primary resistance mutations (PRMs). All had viral load (VL) data at baseline (BL). We report virologic and other outcomes at 12 months (M).

**Results:**

In the overall population, the most common ARTs taken immediately before B/F/TAF were E/C/F/TAF (27%), DTG+F/TAF (9%) and ABC/DTG/3TC (8%). BL genotypic drug resistance testing data were available for 441/996 (44%) participants (ppts); most tests were historic and performed > 60M before starting B/F/TAF (**Table 1**).

Of 441 ppts with BL resistance data, 105/441 (24%) had present/past evidence of PRMs: 13% to NRTI, 11% to NNRTI, 6% to PI, 0.2% to INSTI. The most common PRMs were M184V/I (39 [37%]), ≥ 1 thymidine analog mutation (TAM; 40 [38%]), K103N/S in reverse transcriptase (23 [22%]) and M46I/L in protease (13 [12%]). Primary resistance to > 1 ART drug class was observed in 40 (38%) ppts with PRMs. Ppts with preexisting PRMs were older (≥ 50 years), had more prior ARTs and more prior virologic failure, and had a longer time between HIV diagnosis and starting B/F/TAF versus those without PRMs.

At 12M, effectiveness (HIV-1 RNA < 50 copies/mL; missing VL data = excluded) was maintained in 78 (99%) and 739 (98%) ppts with, versus without, any BL PRMs, respectively; 32/33 (97%) of those with M184V/I alone; 13/13 (100%) with M184V/I + 1–2 TAMs; and 2/2 (100%) with M184V/I + ≥ 3 TAMs at BL. No treatment-emergent PRMs to B/F/TAF were reported. Drug-related adverse events (DRAEs) occurred in 17 (16%) ppts with PRMs versus 113 (13%) without PRMs; serious DRAEs occurred in 2 ppts, both had PRMs at BL. Overall, 98/996 (10%) ppts switched from B/F/TAF to other ARTs (15 [14%] with PRMs, 83 [9%] without PRMs). Additional outcomes are shown in **Table 2**.

**Figure:**
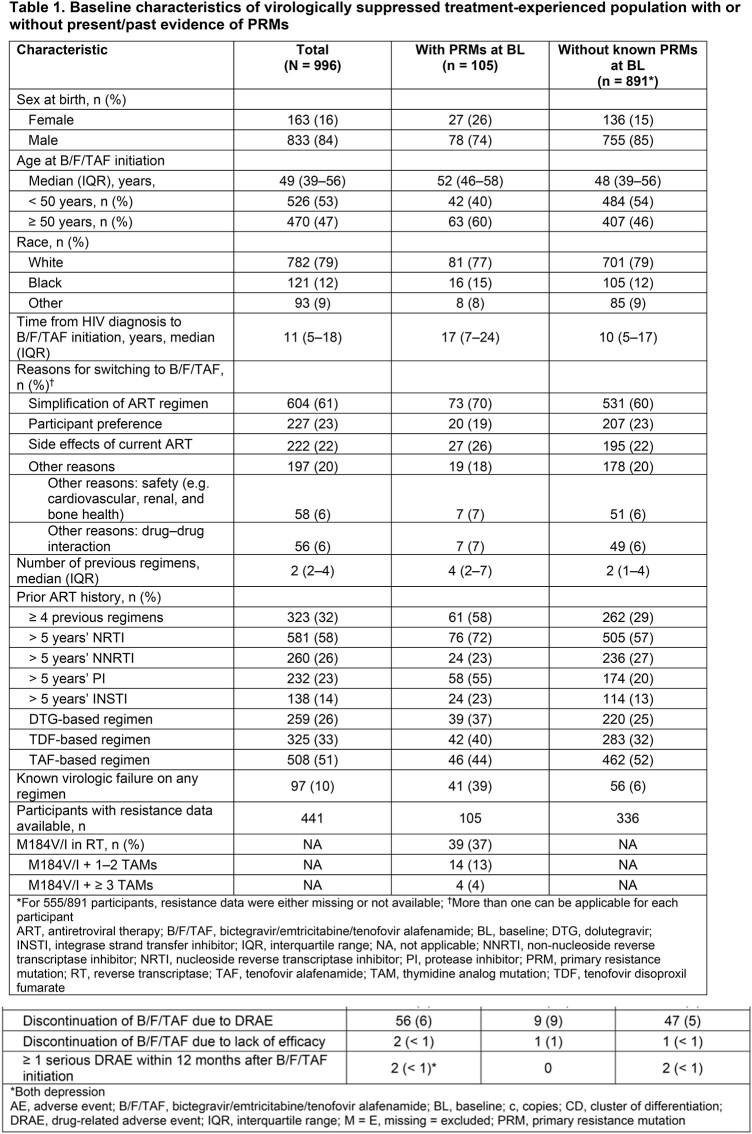

**Figure:**
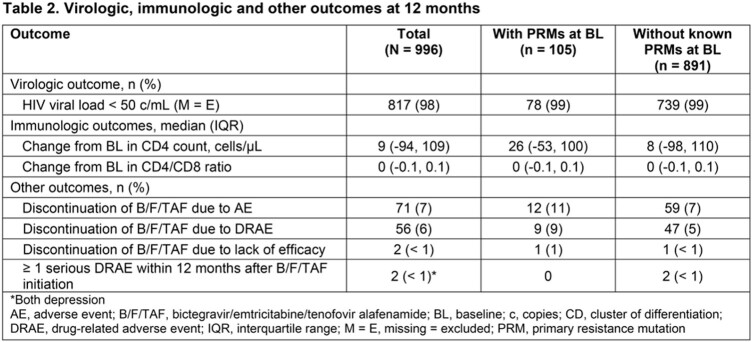

**Conclusion:**

After 12 months, virologically suppressed people with HIV initiating B/F/TAF in routine clinical practice maintained high rates of effectiveness despite the presence of PRMs (including M184V/I).

**Disclosures:**

**Benoit Trottier, MD**, Gilead: Advisor/Consultant|Gilead: Honoraria|Merck: Advisor/Consultant|Merck: Honoraria|ViiV: Advisor/Consultant|ViiV: Honoraria **Fabrice Bonnet, PhD**, Gilead: Grant/Research Support|Gilead: Honoraria|Gilead: Educational **Miguel Garcia-Deltoro, MD, PhD**, AbbVie: Advisor/Consultant|AbbVie: Grant/Research Support|Gilead: Advisor/Consultant|Gilead: Grant/Research Support|Janssen: Board Member|Janssen: Grant/Research Support|MSD: Advisor/Consultant|MSD: Grant/Research Support|ViiV: Advisor/Consultant|ViiV: Grant/Research Support **Massimo Andreoni, MD**, Gilead: Advisor/Consultant|Moderna: Advisor/Consultant|MSD: Advisor/Consultant **Marta Boffito, MD, PhD, FRCP**, AstraZeneca: Honoraria|ATEA: Advisor/Consultant|ATEA: Honoraria|Gilead: Advisor/Consultant|Gilead: Grant/Research Support|Gilead: Honoraria|GSK: Advisor/Consultant|GSK: Grant/Research Support|Moderna: Grant/Research Support|MSD: Advisor/Consultant|MSD: Grant/Research Support|MSD: Honoraria|Novavax: Grant/Research Support|Pfizer: Advisor/Consultant|Pfizer: Grant/Research Support|Roche: Advisor/Consultant|Roche: Grant/Research Support|Valneva: Grant/Research Support|ViiV: Advisor/Consultant|ViiV: Grant/Research Support **Berend J. van Welzen, MD, PhD**, Gilead: Advisor/Consultant|Gilead: Grant/Research Support|ViiV: Advisor/Consultant **Dan Turner, MD**, Gilead: Advisor/Consultant|Gilead: Honoraria|GSK: Advisor/Consultant|GSK: Honoraria **Dai Watanabe, MD, PhD**, Gilead Sciences K.K.: Honoraria|Janssen Pharmaceutical K.K.: Honoraria|MSD K.K.: Honoraria|ViiV Healthcare K.K.: Honoraria **Alper Gündüz, MD**, Gilead: Advisor/Consultant|Gilead: Honoraria|GSK: Advisor/Consultant|GSK: Honoraria|MSD: Advisor/Consultant|MSD: Honoraria **David Thorpe, PhD**, Gilead: Employment|Gilead: Stocks/Bonds **Michelle L. D’Antoni, PhD**, Gilead: Employment|Gilead: Stocks/Bonds **Tali Cassidy, PhD**, Gilead: Employment|Gilead: Stocks/Bonds **Andrea Marongiu, PhD**, Gilead: Employment|Gilead: Stocks/Bonds **Amy R. Weinberg, DNP, MS**, Gilead: Employment|Gilead: Stocks/Bonds **Richard Haubrich, MD**, Gilead: Employment|Gilead: Stocks/Bonds **Stefan Scholten, MD**, AbbVie: Advisor/Consultant|AbbVie: Honoraria|Cepheid: Grant/Research Support|Cepheid: Honoraria|Gilead: Advisor/Consultant|Gilead: Grant/Research Support|Gilead: Honoraria|Gilead: Congress and travel support|GSK: Grant/Research Support|Janssen: Advisor/Consultant|Janssen: Honoraria|Janssen: Congress support|ViiV: Advisor/Consultant|ViiV: Grant/Research Support|ViiV: Honoraria|ViiV: Congress and travel support

